# Correction: Feeding state-dependent regulation of developmental plasticity via CaMKI and neuroendocrine signaling

**DOI:** 10.7554/eLife.11547

**Published:** 2015-09-22

**Authors:** Scott J Neal, Asuka Takeishi, Michael P O'Donnell, JiSoo Park, Myeongjin Hong, Rebecca A Butcher, Kyuhyung Kim, Piali Sengupta

Neal SJ, Takeishi A, O'Donnell MP, Park JS, Hong M, Butcher RA, Kim K, Sengupta P. 2015. Feeding state-dependent regulation of developmental plasticity via CaMKI and neuroendocrine signaling. *eLife* **4**:e10110. doi: 10.7554/eLife.10110

Published 3 September 2015

In the published article, JiSoo Park, Myeongjin Hong and Kyuhyung Kim's affiliation was given as Department of Brain and Cognitive Sciences, Daegu Gyeongbuk Institute of Science and Technology, Daegu, Democratic People's Republic of Korea.

This should have read Department of Brain and Cognitive Sciences, Daegu Gyeongbuk Institute of Science and Technology (DGIST), Daegu, Republic of Korea.

The article has now been corrected.

