# Supplementary material for: Feeding state-dependent regulation of developmental plasticity via CaMKI and neuroendocrine signaling
Source: eLife. 2015 Sep 3;4:e10110. doi: 10.7554/eLife.10110 (PMC4558564; doi:10.7554/eLife.10110)
Supplement: Supplementary file 1. — DOI: http://dx.doi.org/10.7554/eLife.10110.025 [file elife-10110-supp1.docx]

**Table S1.** List of strains used in this work.

| Strain | Genotype | Source and/or  parent strains^a^ | Relevant Figures |
| --- | --- | --- | --- |
| WT | N2 (Bristol) | CGC | 1B, 1C, 1E, 2A, 2D, 3C-D, 4C, 5C-D, 5G, 6E, S1-1A, S1-1C-F, S5-2 |
| CB1112 | *cat-2*(*e1112*) II | CGC | 1C |
| DA521 | *egl-4*(*ad450*) IV | CGC | 1C |
| MT1074 | *egl-4*(*n479*) IV | CGC | 1C |
| MT6308 | *eat-4*(*ky5*) III | CGC | 1C |
| PY8385 | *aak-1*(*tm1944*) III | NBRP | 1C |
| PY8388 | *tph-1*(*mg280*) II | GR1321 | 1C |
| PY8389 | *hlh-30*(*tm1978*) IV | NBRP | 1C |
| RB1588 | *mxl-3*(*ok1947*) X | CGC | 1C |
| RB754 | *aak-2*(*ok524*) X | CGC | 1C |
| SPC168 | *dvIs19* [(pAF15)*gst-4*p::*gfp*::NLS] III; *skn-1*(*lax188*) IV | CGC | 1C |
| YT17 | *crh-1*(*tz2*) III | CGC | 1C |
| PY8386 | *cmk-1*(*oy20*) IV | outcrossed from PY1237 (Satterlee et al., 2004) | 1C-D, S1-1C-D |
| PY8387 | *cmk-1*(*oy21*) IV | outcrossed from PY1589 (Satterlee et al., 2004) | 1C-E, 2A, 2D, 3C-D, 4C, 5D, 5G, 6E, S1-1A, S1-1C-F, S5-2 |
| PY5399 | *cmk-1*(*oy21*) IV; *kyIs128*[*str-3*p::*gfp* *lin-15*+] X; Ex[*gpa-4*p::*cmk-1* *unc-122*p::*dsRed*] | injected into PY1589 | 1E |
| PY5698 | *cmk-1*(*oy21*) IV; *kyIs128*[*str-3*p::*gfp* *lin-15*+] X; Ex[*ttx-1*p::*cmk-1* *unc-122*p::*dsRed*] | injected into PY1589 | 1E |
| PY8390 | *cmk-1*(*oy21*) IV; Ex[*cmk-1*p::*cmk-1* *unc-122*p::*dsRed*] | outcrossed from PY4672 | 1E |
| PY8391 | *cmk-1*(*oy21*) IV; Ex[*sra-9*p::*cmk-1* *unc-122*p::*gfp*] line 1 | injected into PY8387 | 1E |
| PY8392 | *cmk-1*(*oy21*) IV; Ex[*sra-9*p::*cmk-1* *unc-122*p::*gfp*] line 2 | injected into PY8387 | 1E |
| PY8393 | *cmk-1*(*oy21*) IV; Ex[*trx-1*p::*cmk-1* *unc-122*p::*gfp*] line 1 | injected into PY8387 | 1E |
| PY8394 | *cmk-1*(*oy21*) IV; Ex[*trx-1*p::*cmk-1* *unc-122*p::*gfp*] line 2 | injected into PY8387 | 1E |
| PY8395 | *cmk-1*(*oy21*) IV; Ex[*ceh-36*Δp::*cmk-1* *unc-122*p::*dsRed*] line 1 | injected into PY8387 | 1E |
| PY8396 | *cmk-1*(*oy21*) IV; Ex[*ceh-36*Δp::*cmk-1* *unc-122*p::*dsRed*] line 2 | injected into PY8387 | 1E |
| PY8397 | *cmk-1*(*oy21*) IV; Ex[*ceh-36*Δp::*cmk-1* *gpa-4*p::*cmk-1* *unc-122*p::*dsRed*] line 1 | injected into PY8387 | 1E |
| PY8398 | *cmk-1*(*oy21*) IV; Ex[*ceh-36*Δp::*cmk-1* *gpa-4*p::*cmk-1* *unc-122*p::*dsRed*] line 2 | injected into PY8387 | 1E |
| CB1385 | *daf-5*(*e1385*) II | CGC | 2A |
| GR1307 | *daf-16*(*mgDf50*) I | CGC | 2A |
| GR1311 | *daf-3*(*mgDf90*) X | CGC | 2A |
| PY10719 | *cmk-1*(*oy21*) IV; *daf-3*(*mgDf90*) X | PY8387, GR1311 | 2A |
| PY10720 | *daf-5*(*e1385*) II; *cmk-1*(*oy21*) IV | PY8387, CB1385 | 2A |
| PY10721 | *daf-16*(*mgDf50*) I; *cmk-1*(*oy21*) IV | PY8387, GR1307 | 2A |
| FK181 | *ksIs2*[*daf-7*p::*gfp* *rol-6*(*su1006*)] | CGC | 2B-C |
| PY10732 | *cmk-1*(*oy21*) IV; *ksIs2*[*daf-7*p::*gfp* *rol-6*(*su1006*)] | PY8387, FK181 | 2B-C |
| PY10733 | *cmk-1*(*oy21*) IV; *ksIs2*[*daf-7*p::*gfp* *rol-6*(*su1006*)]; Ex[*srg-47*p::*cmk-1* *unc-122*p::*dsRed*] line 1 | injected into PY10732 | 2C |
| PY10734 | cmk-1(oy21) IV; *ksIs2*[*daf-7*p::*gfp* *rol-6*(*su1006*)]; Ex[*srg-47*p::*cmk-1* *unc-122*p::*dsRed*] line 2 | injected into PY10732 | 2C |
| PY10735 | *cmk-1*(*oy21*) IV; *ksIs2*[*daf-7*p::*gfp* *rol-6*(*su1006*)]; Ex[*ceh-36*Δp::*cmk-1* *unc-122*p::*dsRed*] | PY10732, PY8395 | 2C |
| PY10736 | *cmk-1*(*oy21*) IV; Ex[*srg-47*p::*daf-7* *unc-122*p::*gfp*] line 1 | injected into PY8387 | 2D |
| PY10737 | *cmk-1*(*oy21*) IV; Ex[*srg-47*p::*daf-7* *unc-122*p::*gfp*] line 2 | injected into PY8387 | 2D |
| GR1455 | *mgIs40*[*daf-28*p::NLS::*gfp* *lin-15*+] | CGC | 3A-B, S3-1, S5-1 |
| PY10722 | *cmk-1*(*oy21*) IV; *mgIs40*[*daf-28*p::NLS::*gfp* *lin-15*+] | PY8387, GR1455 | 3A-B, S5-1 |
| PY10723 | *cmk-1*(*oy21*) IV; *mgIs40*[*daf-28*p::NLS::*gfp* *lin-15*+]; Ex[*gpa-4*p::*cmk-1* *unc-122*p::*dsRed*] | PY10722, PY5399 | 3B |
| PY10724 | *cmk-1*(*oy21*) IV; *mgIs40*[*daf-28*p::NLS::*gfp* *lin-15*+]; Ex[*trx-1*p::*cmk-1* *unc-122*p::*gfp*] | PY10722, PY8393 | 3B |
| PY10725 | *cmk-1*(*oy21*) IV; *mgIs40*[*daf-28*p::NLS::*gfp* *lin-15*+]; Ex[*ceh-36*Δp::*cmk-1* *unc-122*p::*dsRed*] | PY10722, PY8395 | 3B |
| PY10728 | *cmk-1*(*oy21*) IV; Ex[*srg-47*p::*daf-28*(gDNA) *unc-122*p::*gfp*] line 1 | injected into PY8387 | 3C |
| PY10729 | *cmk-1*(*oy21*) IV; Ex[*srg-47*p::*daf-28*(gDNA) *unc-122*p::*gfp*] line 2 | injected into PY8387 | 3C |
| PY10730 | *cmk-1*(*oy21*) IV; Ex[*trx-1*p::*daf-28*(gDNA) *unc-122*p::*gfp*] line 1 | injected into PY8387 | 3C |
| PY10731 | *cmk-1*(*oy21*) IV; Ex[*trx-1*p::*daf-28*(gDNA) *unc-122*p::*gfp*] line 2 | injected into PY8387 | 3C |
| PY10738 | *daf-28*(*tm2308*) V | NBRP | 3D |
| PY10739 | *cmk-1*(*oy21*) IV; *daf-28*(*tm2308*) V | PY8387, PY10738 | 3D |
| PY8705 | *cmk-1*(*oy21*) IV; Ex[*ttx-1*p::*cmk-1*::*gfp* *unc-122*p::*dsRed*] | (Yu et al., 2014) | 4A |
| PY8906 | Ex[*ceh-36*Δp::*cmk-1*::*gfp* *unc-122*p::*dsRed*] | (3) | 4A-B |
| PY10702 | *cmk-1*(*oy21*) IV; Ex[*ceh-36*Δp::*cmk-1*::NLS::*gfp* *unc-122*p::*gfp*] line 1 | injected into PY8387 | 4C |
| PY10703 | *cmk-1*(*oy21*) IV; Ex[*ceh-36*Δp::*cmk-1*::NLS::*gfp* *unc-122*p::*gfp*] line 2 | injected into PY8387 | 4C |
| PY10704 | *cmk-1*(*oy21*) IV; Ex[*ceh-36*Δp::*cmk-1*::NES::*gfp* *unc-122*p::*gfp*] line 1 | injected into PY8387 | 4C |
| PY10705 | *cmk-1*(*oy21*) IV; Ex[*ceh-36*Δp::*cmk-1*::NES::*gfp* *unc-122*p::*gfp*] line 2 | injected into PY8387 | 4C |
| LRB50 | *unc-119*(*ed9*) III; Ex[*ins-26*p::*yfp*::PEST *unc-119*+] | (Chen and Baugh, 2014) | 5A-B |
| LRB54 | *unc-119*(*ed9*) III; Ex[*ins-35*p::*yfp*::PEST *unc-119*+] | (Chen and Baugh, 2014) | 5A-B |
| PY10706 | *cmk-1*(*oy21*) IV; Ex[*ins-26*p::*yfp*::PEST *unc-119*+] | PY8387, LRB50 | 5A-B |
| PY10707 | *cmk-1*(*oy21*) IV; Ex[*ins-35*p::*yfp*::PEST *unc-119*+] | PY8387, LRB54 | 5A-B |
| PY10804 | *cmk-1*(*oy21*) IV; Ex1[*ins-35*p::*yfp*::PEST *unc-119*+]; Ex2[*ceh-36*Δp::*cmk-1*+NLS *unc-122*p::*dsRed*] line 1 | injected into PY10707 | 5B |
| PY10805 | *cmk-1*(*oy21*) IV; Ex1[*ins-35*p::*yfp*::PEST *unc-119*+]; Ex2[*ceh-36*Δp::*cmk-1*+NLS *unc-122*p::*dsRed*] line 2 | injected into PY10707 | 5B |
| PY10806 | *cmk-1*(*oy21*) IV; Ex1[*ins-35*p::*yfp*::PEST *unc-119*+]; Ex2[*ceh-36*Δp::*cmk-1*+NES *unc-122*p::*dsRed*] line 1 | injected into PY10707 | 5B |
| PY10807 | *cmk-1*(*oy21*) IV; Ex1[*ins-35*p::*yfp*::PEST *unc-119*+]; Ex2[*ceh-36*Δp::*cmk-1*+NES *unc-122*p::*dsRed*] line 2 | injected into PY10707 | 5B |
| PY7502 | *oyIs85*[*ceh-36*Δp::TU813(*recCaspase*) *ceh-36*Δp::TU814(*recCaspase*) *unc-122*p::*dsRed* *srtx-1*p::*gfp*] | (Beverly et al., 2011) | 5C, 5F, 5G |
| PY10712 | *ins-26*(*tm1983*) I; *ins-35*(*ok3297*) V | PY10708, RB2412 | 5C-D |
| PY10808 | *ins-32*(*tm6109*) II | NBRP | 5C-D |
| PY10708 | *ins-26*(*tm1983*) I | NBRP | 5D |
| PY10709 | *ins-26*(*tm1983*) I; *cmk-1*(*oy21*) IV | PY8387, PY10708 | 5D |
| PY10710 | *ins-35*(*ok3297*) V | outcrossed from RB2412 | 5D |
| PY10711 | *cmk-1*(*oy21*) IV; *ins-35*(*ok3297*) V | PY8387, RB2412 | 5D |
| PY10713 | *cmk-1*(*oy21*) IV; Ex[*che-1*p::*ins-26* *che-1*p::*ins-35* *unc-122*p::*gfp*] line 1 | injected into PY8387 | 5D |
| PY10714 | *cmk-1*(*oy21*) IV; Ex[*che-1*p::*ins-26* *che-1*p::*ins-35* *unc-122*p::*gfp*] line 2 | injected into PY8387 | 5D |
| PY10715 | *cmk-1*(*oy21*) IV; Ex[*trx-1*p::*ins-26* *trx-1*p::*ins-35* *unc-122*p::*gfp*] line 1 | injected into PY8387 | 5D |
| PY10716 | *cmk-1*(*oy21*) IV; Ex[*trx-1*p::*ins-26* *trx-1*p::*ins-35* *unc-122*p::*gfp*] line 2 | injected into PY8387 | 5D |
| PY10717 | *cmk-1*(*oy21*) IV; Ex[*ceh-36*Δp::*ins-26* *ceh-36*Δp::*ins-35* *unc-122*p::*gfp*] line 1 | injected into PY8387 | 5D |
| PY10718 | *cmk-1*(*oy21*) IV; Ex[*ceh-36*Δp::*ins-26* *ceh-36*Δp::*ins-35* *unc-122*p::*gfp*] line 2 | injected into PY8387 | 5D |
| PY10809 | *ins-32*(*tm6109*) II; *cmk-1*(*oy21*) IV | PY8387, PY10808 | 5D |
| PY10726 | *cmk-1*(*oy21*) IV; *mgIs40*[*daf-28*p::NLS::*gfp* *lin-15*+]; Ex[*ceh-36*Δp::*ins-26* *ceh-36*Δp::*ins-35*::SL2::*mCherry* *unc-122*p::*dsRed*] line 1 | injected into PY10722 | 5E |
| PY10727 | cmk-1(oy21) IV; *mgIs40*[*daf-28*p::NLS::*gfp* *lin-15*+]; Ex[*ceh-36*Δp::*ins-26* *ceh-36*Δp::*ins-35*::SL2::*mCherry* *unc-122*p::*dsRed*] line 2 | injected into PY10722 | 5E |
| PY10700 | *cmk-1*(*oy21*) IV; *oyIs85*[*ceh-36*Δp::TU813(*recCaspase*) *ceh-36*Δp::TU814(*recCaspase*) *unc-122*p::*dsRed* *srtx-1*p::*gfp*] | PY8387, PY7502 | 5G |
| PY7548 | Ex[*ceh-36*Δp::*GCaMP3* *unc-122*p::*dsRed*] | (Beverly et al., 2011) | 6A-D, S6-1 |
| PY8399 | *cmk-1*(*oy21*) IV; Ex[*ceh-36*Δp::*GCaMP3* *unc-122*p::*dsRed*] | PY8387, PY7548 | 6A-D, S6-1 |
| PY9232 | Ex[*ceh-36*Δp::*HisCl1*::SL2::  *mCherry* *unc-122*p::*gfp*] | (Pokala et al., 2014) | 6E |
| PY10810 | *cmk-1*(*oy21*) IV; Ex[*ceh-36*Δp::*HisCl1*::SL2::  *mCherry* *unc-122*p::*gfp*] | PY8387, PY9232 | 6E |
| PY10813 | Ex[*ceh-36*Δp::*twk-18(gf)* *unc-122*p::*gfp*] line 1 | injected into WT | 6F |
| PY10814 | Ex[*ceh-36*Δp::*twk-18(gf)* *unc-122*p::*gfp*] line 2 | injected into WT | 6F |
| PY10815 | *cmk-1*(*oy21*) IV; Ex[*ceh-36*Δp::*twk-18(gf)* *unc-122*p::*gfp*] line 1 | injected into PY8387 | 6F |
| PY10816 | *cmk-1*(*oy21*) IV; Ex[*ceh-36*Δp::*twk-18(gf)* *unc-122*p::*gfp*] line 2 | injected into PY8387 | 6F |
| DR476 | *daf-22*(*m130*) II | CGC | S1-1B |
| PY1991 | *lin-15*(*n765ts*); (Ex[*cmk-1*p::*gfp* *lin-15+*] | (Satterlee et al., 2004) | S1-2 |
| PY10740 | Ex[*ceh-36*Δp::*cmk-1*::NLS::*gfp* *unc-122*p::*gfp*] | injected into WT | S4-1A |
| PY10741 | Ex[*ceh-36*Δp::*cmk-1*::NES::*gfp* *unc-122*p::*gfp*] | injected into WT | S4-1A |
| PY10742 | *cmk-1*(*oy21*) IV; Ex[*ceh-36*Δp::*cmk-1*::NLS::*gfp* *unc-122*p::*gfp*] | injected into PY8387 | S4-1B |
| PY10743 | *cmk-1*(*oy21*) IV; Ex[*ceh-36*Δp::*cmk-1*::NES::*gfp* *unc-122*p::*gfp*] | injected into PY8387 | S4-1B |
| PY10811 | *mgIs40*[*daf-28*p::NLS::*gfp* *lin-15*+]; *oyIs85*[*ceh-36*Δp::  TU813(*recCaspase*) *ceh-36*Δp::TU814(*recCaspase*) *unc-122*p::*dsRed* *srtx-1*p::*gfp*] | GR1455, PY7502 | S5-1 |
| PY10812 | *cmk-1*(*oy21*) IV; *mgIs40*[*daf-28*p::NLS::*gfp* *lin-15*+]; *oyIs85*[*ceh-36*Δp::TU813  (*recCaspase*) *ceh-36*Δp::  TU814(*recCaspase*) *unc-122*p::*dsRed* *srtx-1*p::*gfp*] | PY10722, PY10700 | S5-1 |
| PY10744 | *bli-4*(*e937*) I; *cmk-1*(*oy21*) IV | PY8387, CB937 | S5-2 |
| PY10745 | *cmk-1*(*oy21*) IV; Ex[*ceh-36*Δp::*bli-4*(S)::SL2::*mCherry* *ceh-36*Δp::*bli-4*(AS)::SL2::*mCherry* *unc-122*p::*gfp*] line 1 | injected into PY8387 | S5-2 |
| PY10746 | *cmk-1*(*oy21*) IV; Ex[*ceh-36*Δp::*bli-4*(S)::SL2::*mCherry* *ceh-36*Δp::*bli-4*(AS)::SL2::*mCherry* *unc-122*p::*gfp*] line 2 | injected into PY8387 | S5-2 |
| PY10747 | *cmk-1*(*oy21*) IV; Ex[*odr-1*p::*bli-4*(S)::SL2::*mCherry* *odr-1*p::*bli-4*(AS)::SL2::*mCherry* *unc-122*p::*gfp*] line 1 | injected into PY8387 | S5-2 |
| PY10748 | *cmk-1*(*oy21*) IV; Ex[*odr-1*p::*bli-4*(S)::SL2::*mCherry* *odr-1*p::*bli-4*(AS)::SL2::*mCherry* *unc-122*p::*gfp*] line 2 | injected into PY8387 | S5-2 |
| PY10749 | *cmk-1*(*oy21*) IV; Ex[*odr-3*p::*bli-4*(S)::SL2::*mCherry* *odr-3*p::*bli-4*(AS)::SL2::*mCherry* *unc-122*p::*gfp*] line 1 | injected into PY8387 | S5-2 |
| PY10750 | *cmk-1*(*oy21*) IV; Ex[*odr-3*p::*bli-4*(S)::SL2::*mCherry* *odr-3*p::*bli-4*(AS)::SL2::*mCherry* *unc-122*p::*gfp*] line 2 | injected into PY8387 | S5-2 |
| PY10751 | *cmk-1*(*oy21*) IV; Ex[*ceh-36*Δp::*egl-3*(S)::SL2::*mCherry* *ceh-36*Δp::*egl-3*(AS)::SL2::*mCherry* *unc-122*p::*gfp*] line 1 | injected into PY8387 | S5-2 |
| PY10752 | *cmk-1*(*oy21*) IV; Ex[*ceh-36*Δp::*egl-3*(S)::SL2::*mCherry* *ceh-36*Δp::*egl-3*(AS)::SL2::*mCherry* *unc-122*p::*gfp*] line 2 | injected into PY8387 | S5-2 |
| PY10753 | *cmk-1*(*oy21*) IV; Ex[*odr-1*p::*egl-3*(S) *odr-1*p::*egl-3*(AS) *unc-122*p::*gfp*] | PY8387, ZC2114 (Harris et al., 2014) | S5-2 |

^a^CGC – *Caenorhabditis* Genetics Center; NBRP – National BioResource Project

**REFERENCES**

BEVERLY, M., ANBIL, S. & SENGUPTA, P. 2011. Degeneracy and signaling within a sensory circuit contributes to robustness in thermosensory behaviors in *C. elegans*. *J Neurosci,* 31**,** 11718-11727.

CHEN, Y. & BAUGH, L. R. 2014. *ins-4* and *daf-28* function redundantly to regulate *C. elegans* L1 arrest. *Dev Biol,* 394**,** 314-26.

HARRIS, G., SHEN, Y., HA, H., DONATO, A., WALLIS, S., ZHANG, X. & ZHANG, Y. 2014. Dissecting the signaling mechanisms underlying recognition and preference of food odors. *J Neurosci,* 34**,** 9389-403.

POKALA, N., LIU, Q., GORDUS, A. & BARGMANN, C. I. 2014. Inducible and titratable silencing of *Caenorhabditis elegans* neurons in vivo with histamine-gated chloride channels. *Proc Natl Acad Sci USA,* 111**,** 2770-5.

SATTERLEE, J. S., RYU, W. S. & SENGUPTA, P. 2004. The CMK-1 CaMKI and the TAX-4 cyclic nucleotide-gated channel regulate thermosensory neuron gene expression and function in *C. elegans*. *Curr Biol,* 14**,** 62-8.

YU, Y. V., BELL, H. W., GLAUSER, D. A., GOODMAN, M. B., VAN HOOSER, S. D. & SENGUPTA, P. 2014. CaMKI-dependent regulation of sensory gene expression mediates experience-dependent plasticity in the operating range of a thermosensory neuron. *Neuron,* 84**,** 919-926.
